# Account of Ann Moor

**Published:** 1810-10

**Authors:** Richard Thompson

**Affiliations:** Birmingham


### *To the Editor of the Medical and Physical Journal.*

SIR,

EDUCATED in the public schools, where system—that stepping stone to knowledge, hold an unresisted, undivided sway; I have been taught to think, that the natural world is governed by laws uniform and immutable. Nor is this doctrine in the least at variance with reason. If we look around us, every object forces itself upon our thoughts as the creature of design; we see, and we admire the regularity with which the same laws produce the infinite variety of effects which characterize the operations of nature. The power which we call gravitation, the living principle, electricity, and other natural causes produce the same effects; and although there seem to exist many anomalies in the action of these laws, yet their number is daily on the decline. Children in science, we find, that as our knowledge increases, this beautiful uniformity becomes more apparent; a circumstance which materially strengthens the belief, that if the various phænomena presented to us were properly understood, we should find one grand law to precide over every change in the material world.

I have been led into this train of thought from the perusal of various papers which have recently appeared in the Journals, on the subject of a woman named Ann Moor, at present residing at Tutbury, in Staffordshire, who is supposed by the people in that neighbourhood, and by several medical men, to have lived for two years without having taken any kind of food.

Reasoning *a priori* upon this circumstance, I should as soon expect that a body projected from the earth would not return as that it should be true; I shall venture, therefore, to offer you a few observations upon the case:

It is certain that we find occasional instances of animals being variously deformed, but there is some, at least, that I have met with, in which there has been the deficiency of a stomach. Indeed, in the myriads of living objects with which the face of nature teems, there is not a single species in which this organ is wanting—an hydatide, the most simply constructed creature creature that exists, is all stomach; and it will be found, that as animals become more complicated in their organization, it is always by the addition of other apparatus to this indispensable appendage. Reasoning in this way, we should be very cautious in giving credit to any alledged instance of the stomach becoming useless, especially when there is no other apparent route, by which the aliment can be supplied to the constitution. Certainly, in cases of fever and other illness, patients will live for weeks without food; but although these persons in every case take liquids, they invariably become emaciated, and would die, if the abstinence were continued. Here too, the body is in a very different state from that of health; the pulse is quick, and every office is more or less deranged.

How different, however, is the case of Ann Moore. *Her* appearance is that of a handsome, respectable-looking woman, who is beginning to wear her grey hairs with proper dignity. She is pale indeed, but looks healthy; and so far from the functions of her body being deranged, there is not one* which does not go on with the regularity of health. Her pulse is under 80, firm and good; her strength very little impaired; her conversation correct; the tone of her voice remarkably full and strong; her perspiration free and natural; and *her mouth perfectly supplied with saliva; by her own account,* she *passes urine sometimes;* has the motion of all her limbs, and certainly is not emaciated to any great extent, at all events, not more so than would be expected in any one who has been so long confined to bed. This is a curious state for a person to be in who has neither eaten nor drank for two years.

Not to mention the indications of unimpaired strength which she possesses, let us for a moment consider the state of her excretions. The quantity of fluid which is perspired by a human subject, is determined by Lavoisier and Sequin, amounts to 17.63 grains in the minute, or 52.89 ounces in the 24 hours. This fact alone, when applied to the case under examination, is sufficient to shake the firmest belief of it. The woman's own account with regard to her urine is, I believe, that she makes about a tea-cupful once in a week. Even if she made no more, which is much to be doubted, her accounts to different people being very contradictory, whence is this derived? A medical friend of mine, to whom she said she had passed urine within two or three days of the time he saw her, could plainly perceive the bladder above the pubis, to contain a con-

* Those I mean which she cannot, or thinks it unnecessary to disguise.

siderably siderably larger quantity than that she mentioned, so much so, indeed, as to convince him, her own statement as to that point, was absolutely false.

But the most remarkable circumstance with regard to her relates to the secretion of saliva. It is now generally agreed upon by physiologists, that the great use of this secretion is to act an important part in the process of digestion; and in proof of this, we find, that whenever that operation is deranged, the state of the mouth is an instant and accurate test of the fact. In this case, however, the tongue is tolerably clean, and the saliva neither diminished in quantity, nor vitiated in quality. This is an unanswerable proof that she does take food in certain, although probably in very small quantities. Nor is this at all an unusual circumstance. The most inattentive observer must have seen abundance of instances, in which women have existed, and seemingly in very tolerable health, upon almost inconceivably small quantities of food; and it is impossible to say, to what extent this might be carried, especially where there is chronic disease of the viscera, which is perhaps the case with this woman.

From such considerations, it is my decided opinion that she is an impostor; and in farther corroboration, I shall now mention the result of an interview with her, which took place some time ago. She seemed to be in a state of weakness, which made it great labour, as well as pain, for her even to attempt to move. This but ill agreed with her countenance and pulse. We carried on the conversation for some time, in which she took a lively part, till we began to insinuate that the medical men of London were so dissatisfied with the manner in which her case had been proved by watching, that we had heard it was the intention of a certain number of them to come down, for the purpose of watching her themselves, which would be so managed, that at no time should she be without the eyes of a medical man being upon her. At this suggestion she took great offence, and in the course of a little time, so completely forgot her situation, that she raised herself upright in bed, a position which we had *previously* learned she had not been in for more than a year, griped her fists, threw her arms and head about with as much strength and ease as the most healthy woman of an equal age could possibly do, and talked at the same time most loudly and incessantly, from the effect of violent passion. She complained of our cruelty to her bitterly, and said we meant to impose upon her; that she had been upon her trial once, which she would not then have submitted to, but to oblige the *Minister*, and for nobody in the world would she undergo undergo a repetition of it*. So convinced were we now of her true character, that after paying the admittance fee, we immediately left her; and the impression our interview made upon her was so lasting, that she continues to this day to tell the people that visit her of the circumstance, which she converts to good account, by saying, that we intended *to frighten her*, but she was *fully aware* of our *designs.*

That the public may understand the minutiæ of this case, it is right to state, that she is of the puritanical profession of faith, and strongly impresses upon the minds of her visitors that her case is a miracle. However this may be, she has made it answer very well in a pecuniary point of view, which it is much to be suspected was the chief end she wished to gain. What makes this more probable is, that for some time past, I understand she has told every new comer that "God is able to restore her to her former health, and that very probably he will do it." With respect to the description of people who watched her during the time of her probation, it is enough to state, that they were all belonging to the town or neighbourhood of Tutbury, most of them her own acquaintance, and of the same religious persuasion as herself. Although miracles are supposed to have ceased long since, it would seem as if the neighbourhood of Tutbury was either very highly favoured by the Almighty, or its inhabitants particularly sceptical in their religious opinions; for since this woman has derived so much advantage from her infirmities, another miracle has started at Barton, near Tutbury, in the person of a boy, who, it is said, can only see on Sundays, being blind all the rest of the week. This I only know from report, but have every reason to give credit to it.

On reading the Edinburgh Medical and Surgical Journal, I find a paper upon this person's case, by Mr. Granger, of Burton-on-Trent, near Tutbury, and am sorry to see our accounts differ so much, as upon comparison I find is the case; and yet, upon re-examination, I see the great difference between us is, in those circumstances for which our only proof is her own account, with the exception of the state of her pulse, which may be different at different times, the great sinking of the abdomen and emaciation of the legs, neither of which I thought extraordinary for a person who had been in bed for two years.

The cases which Mr. Granger relates in corroboration of his account, I cannot bring myself to see in any light but that of

* Her attendant, who is as well an educated hypocrite as her mistress, was pleased to style it "*a trial for her life.*" This she did in arguing against her mistress submitting to a second.

impostors.

impostors. Of these, I shall mention that of Janet M'Leod, who after four years abstinence had, "her features clear and not disfigured or sunk; her breast round and prominent like those of a healthy young woman; her legs, arms, and thighs, not at all emaciated." Her cure was uncommonly sudden and complete; for, "all at once she cried out for a drink, and drank a pint of water at one draught." That also of "a young and beautifu woman who fasted 34 days, and soon after 54 days, without having lost much of her flesh." That also of an abstinence of 54 *years* from the Edinburgh Med. Essays, where the patient, "only voided fœces *once a year*, and that *always* in the *month* of *March*." These cases bear internal marks of their being void of truth, and as such, require no observation.

I think Mr. Granger has erred in taking them all for granted, without considering whether the great authors, who have detailed them, *might* not as well have been deceived by a cunning woman and her accomplices, as many of the too credulous people in the neighbourhood of Ann Moore,

RICHARD THOMPSON.

*Birmingham*,
*August* 7, 1809.

